# Recovery from musculoskeletal injury: the role of social support following a transport accident

**DOI:** 10.1186/s12955-015-0291-8

**Published:** 2015-07-03

**Authors:** Khic-Houy Prang, Janneke Berecki-Gisolf, Sharon Newnam

**Affiliations:** Monash Injury Research Institute, Monash University, Melbourne, Victoria Australia

**Keywords:** Social support, Musculoskeletal injury, Injury outcomes

## Abstract

**Background:**

Social support can be an important coping resource for persons recovering from injury. In this study, we examined the effects of family structure and sources of social support on physical health, persistent pain and return to work (RTW) outcomes following musculoskeletal injury (MSI) sustained in a transport accident.

**Methods:**

Secondary analysis of Transport Accident Commission (TAC) cross-sectional surveys held in 2010 and 2011 was conducted. In total 1649 persons with MSI were identified and included. Family structure was determined by marital status and number of children. Sources of social support were measured as perceived help from family, friends, neighbours and employers. Physical health was measured with the Physical Component Summary (PCS) score of the Short-Form-12 Health Survey Version 2. Persistent pain was defined as self-reported persistent pain experienced in the last 3 months, and RTW was defined as being back at work for ≥3 months at time of interview. Multiple linear and logistic regressions were used for the analyses.

**Results:**

Family and friends’ support was associated with better physical health among persons with >1 day hospital stay. Being married or in a de facto relationship was associated with greater PCS score among non-hospitalised persons. Being widowed/separated/divorced was associated with more self-reported persistent pain (odds ratio 1.62 [95 % confidence intervals 1.11–2.37]). Support from family (0.40 [0.24–0.68]), friends (0.29 [0.17–0.47]) and neighbours (0.59 [0.41–0.84]) was associated with less persistent pain. Among women, support from family (0.09 [0.01–0.78]) was negatively associated with RTW, whereas support from friends (3.03 [1.15–8.02]) was positively associated with RTW. These associations were not observed among men. For both men (5.62 [2.77–11.38]) and women (7.22 [2.58–20.20]), support from employers was positively associated with RTW.

**Conclusion:**

Family structure and sources of social support had a positive impact on physical health, persistent pain and RTW following MSI. This study highlights the importance of identifying people who have limited access to a social support network. Those with limited access to social support after a transport accident could potentially benefit from the provision of formal sources of practical and psychological support.

## Background

Musculoskeletal injuries (MSI) are the most common type of injury sustained [1] and source of morbidity following a transport accident [2]. The Global Burden of Disease study showed that the majority of admissions for various non-fatal injuries as a result of a transport accident are MSI. Almost 50 % of non-fatal injuries sustained were fractures [3]. During the 2012/13 fiscal year, in Victoria, Australia, 4031 people were admitted to hospital and 4787 people visited an emergency department for MSI sustained in a transport accident. The most common MSI reported in Victorian hospitals were fractures to the thorax, shoulder and upper arm, knee and lower leg, followed by dislocation, sprain and strain to the neck, shoulder and upper arm [4].

Recovery from MSI sustained in a transport accident varies considerably from rapid and complete recovery to substantial delayed recovery with symptoms persisting for several years. For example, studies conducted by Ottoson and colleagues [5] and Littleton and colleagues [6] found that 60 % of people return to their pre-injury level of physical health at 6 months following minor MSI sustained in transport accident. Littleton and colleagues also found that after 6 months significant improvement of physical health ceased [6]. Similarly, other longitudinal studies reported that at 1 to 3 years post-injury, 20–40 % of people continued to experience poor recovery and had not returned to their pre-injury level of physical health [7–9]. Severe pain and psychiatric problems resulting from MSI have also been reported in the literature [10]. Due to varying rates of recovery following injury, there is a need to identify risk factors associated with poor outcomes.

Variability in health outcomes post-MSI is influenced by a number of behavioural and psychosocial factors [11, 12], including social support [13]. Social support is defined as information leading individuals to believe they are cared for and loved, esteemed and valued and belong to a network of communication and mutual obligation [14]. Much literature has shown that social support is associated with promoting good physical and mental health [15, 16], reducing and preventing illness [17], and moderating life stress [18]. Conversely, the absence of social support (i.e., social deprivation) or poor social support can result in substantial health risk including an increase in psychiatric morbidity [19] and mortality [20].

In regards to the the relationship between social support and MSI outcomes, the evidence has been mixed. There is evidence that social support is associated with better functional outcomes [21], lower pain intensity [22], and return to work (RTW) [23]. Lack of social support or perceived negative support has also been associated with the development of post-traumatic stress disorder [19, 24]. In contrast, a recent systematic review found inconclusive evidence of an association between social support and MSI outcomes [25].

It is possible that the inconclusive evidence from the systematic review could be attributed to limitations in the methodology and conceptual design of previous research studies. In regards to the methodological limitations, previous studies have focused on only one health outcome [19, 24, 26]. Comparing results across a range of injury outcomes within the same population can provide more robust evidence regarding the differential impact of social support on injury outcomes. In addition, samples have included all injuries sustained in a transport accident regardless of injury types and severity [19, 24, 27]. It is possible that different types of social support may be required depending on the type and severity of the injury. These methodological limitations will be addressed in this study by exploring various outcomes among a sample with MSI.

Past research has also been limited in its conceptual development. Studies have focused on a single dimension of social support without specifying the source of social support [19, 21, 22, 26]. Social support is a multi-dimensional construct and both the structural and functional aspects of support should be measured. The majority of the literature suggests that social support facilitates recovery [21–23], but it is also possible that poor recovery leads to a deterioration of social support networks (reverse causality). To date, the causal pathway is generally assumed to run unidirectionally from social support to health. To address these conceptual limitations, we explore elements of the convoy model [28] to better understand the impact of social support on recovery from MSI. This model will allow us to more fully understand how social relations between the individual and each member of the convoy model vary across injury outcomes.

### The convoy model

According to the convoy model*,* social support varies in terms of structural support, functional support and adequacy [28]. Structural support refers to the characteristics of the network of people surrounding an individual over the life course and his/her interaction with this network (e.g., network size, marital status). Functional support describes the aid and encouragement that is provided to the individual by the social network. Types of functional support include informational (e.g., providing guidance), tangible (e.g., help getting tasks done), appraisal (e.g., help evaluating a situation) and emotional support (e.g., feelings of being loved) [28–30]. Adequacy is defined as the level to which the individual finds the support they are providing or receiving adequate. Furthermore, the convoy model suggests that social support and social relations are shaped by personal (e.g., age, gender) and situational characteristics (e.g., life events); the combination of which influences individual health and well-being [28].

Social support can originate from a variety of sources including informal (e.g., spouse, family, friends) or formal sources (e.g., healthcare professionals). According to the convoy model, individuals are also surrounded by close social relationships at various stages of the life span [28]. Generally, the convoy model has been represented as a concentric-circle diagram in which the circles are used to separate people in terms of the closeness of their relationship with an individual.

This model provides a framework for exploring the role of social support in recovery from MSI. The convoy model proposes that the structural, functional and adequacy aspects of social support is influenced by a person’s traits and demands of the context; yet to our knowledge these assumptions, particularly in relation to recovery from MSI are yet to be established in the research literature. The aim of the present study is to determine the impact of social support within the social network (family, friends, neighbours and employer support) on injury outcomes (physical health, persistent pain and RTW) following compensable MSI. In particular, this study will examine the family structure and sources (family, friends, neighbours, and workplace) of social support that have the greatest impact on recovery.

## Methods

### Study design

The study involved secondary analysis of de-identified cross-sectional surveys conducted among compensated transport accident victims in Victoria, Australia 2010 and 2011.

### Transport injury compensation system

In the state of Victoria, Australia, those injured in land-based transport accidents involving a car, motorcycle, tram, bus or train are eligible to claim compensation for treatment, income replacement, rehabilitation and long-term support services via the Transport Accident Commission (TAC), regardless of fault. In addition, the TAC provides compensation for injury and death occurring interstate for individuals travelling in a Victorian-registered motor vehicle in other Australian states. Injuries and death occurring on the road but not involving a motorised vehicle (e.g., a collision between a pedal cyclist and a pedestrian) are not eligible for compensation (www.tac.vic.gov.au).

### Data source

The TAC conducts an annual Client Outcomes Survey (COS) measuring the health and vocational status of its clients. The survey was designed to inform the TAC about the impact of its claims management practices and the design of the compensation scheme on the health and vocational outcomes of its clients. The survey includes standardised measures of vocational and health status prior to injury, current vocational status, current health status, including physical and mental health, persistent pain, mobility and functional independence, access to and satisfaction with healthcare and satisfaction with the TAC.

The sample was randomly selected from the population of TAC in the Recovery branch. Clients in the Recovery branch generally have more severe injuries, complex recoveries and require longer-term support. In 2010 and 2011, 6559 and 7739 clients records were provided by the TAC to a third party social research organisation, respectively. The large sample provided was to ensure sufficient sample to fill all required quotas and allow for the opt-out process. Approach letters and TAC research program brochures introducing the survey were mailed to clients. Clients could then either opt-in or opt-out of the study by calling the contact number included in the letter. Clients who took no action were included in the study. The required sample size for 2010 and 2011 were 1290 and 1165 clients, respectively. Subgroups quotas were set by the TAC against teams, life of claim (~4 months to 6 years*)* as well as active and inactive claims. Active claims were defined as a payment received from the TAC within the last 6 months prior to being surveyed. Inactive claims were defined as no active payments made in the last 6 months, but at least one payment made within 7 to 24 months prior to being surveyed. Once a subgroup quota has been achieved, the third party social research organisation no longer interviewed individual for that particular subgroup. If quota groups appeared to have lower response numbers than expected, efforts were made to target clients in these groups. Data was collected via computer automated telephone interview (CATI) conducted by a third-party social research organisation. The questionnaire takes approximately 25 minutes to administer.

### Study participants

In 2010 and 2011, 5266 participants were approached to participate in the study. Of these, 2476 participants completed the survey, including 1649 (67 %) participants with MSI. The survey participation rates for 2010 and 2011 were 44 % and 46 %, respectively. The study comprised of 60 % active claims and 40 % inactive claims. The sample comprised of minor to moderate injuries such as soft tissue, complex orthopaedic/multi-trauma including mild and moderate brain injury. Catastrophic injuries such as spinal cord injury, severe traumatic brain injury, amputees and burns were excluded. In this study, the sample was limited to participants with MSI including sprains/strains, soft tissues, fractures and dislocations.

### Measures

The measures used in this study are part of the standard COS.

#### Demographics and injury-related characteristics

Demographic characteristics included gender (female vs. male), age, country of birth (Australia vs. others), education (university level vs. less than university level), residential location (Melbourne vs. rest of Victoria), employment status at time of accident (yes vs. no), occupation and income (less than $50,000 vs. more than $50,000). Age was defined as the age of the participant at the time of the interview. Among those working at the time of the accident, occupation was grouped into 8 groups: managers, professionals, technicians and trade workers, community/personal service workers, clerical/administration workers, sales workers, machine operators/drivers and labourers [31]. Injury-related characteristics included pre-injury health status (excellent, very good, good, fair, poor), injury type (soft tissue including whiplash, sprain and strain, fracture, dislocation) and hospitalisation (>1 day hospital stay vs. not hospitalised) which was used as a proxy for injury severity [32, 33]. Time since injury was derived from the date of the interview and the accident date.

#### Social support

Family structure included marital status and number of dependent children. Marital status was grouped into married/de facto relationship, widowed/separated/divorced and never married. As expected, preliminary analysis showed an association between marital status and number of dependent children; thus a family structure composite variable was created. The family composition was categorised into 6 groups: married/de facto relationship with children, married/de facto relationship with no children, widowed/separated/divorced with children, widowed/separated/divorced with no children, never married with children, never married with no children. Sources of social support included accessing help from family, friends, neighbours and employers. For family, friends and neighbours items, participants rated their level of agreement using a 4-point scale that ranged from 1 *yes, definitely* to 4 *no, not at all* to the following question; *‘Can you get help from family members/friends/neighbours if you need it?’*. For the employer’s item, participants also rated their level of agreement using a 4-point scale that ranged from 1 *very supportive* to 4 *not at all supportive* to the following question*; ‘Thinking about the time you had off work following your accident, in general how supportive was your employer?’*.

#### Physical health

Physical health was assessed by the Short-Form-12 Health Survey Version 2 (SF-12 V2). The SF-12 V2 is a validated international tool that consists of twelve questions [34]. The Physical Component Summary (PCS) of the SF-12 V2 focuses mainly on limitations in physical functioning, role limitations due to physical health problems, bodily pain, and general health. The PCS scores were derived using Australian weights based on the Australian population norms [35]. Higher scores on the PCS indicated more positive physical health.

#### Persistent pain

Persistent pain was used as an indicator of recovery and was defined as pain that lasted for at least 3 months, as a result of the injury (at the time of the survey). Participants responded yes/no to the following question; ‘*Has this pain last more than 3 months?’* However, this does not mean that participant had to be in pain every day over the last 3 months; rather, they experienced pain on most days during this time, whether it be continual or not.

#### Return to work (RTW)

RTW outcome was also an indicator of recovery. RTW was defined as those who had time off work as a result of their accident but who had been back at work for 3 months or more and those who had returned to work initially but who had ceased working for reasons unrelated to their accident. RTW was coded as yes/no and was derived from the following questions: ‘*Did you have a paid job of any kind at the time of the accident?’, ‘Did you take any time off work as a result of your accident?’, ‘How long have you been back at work in this job?’, ‘Thinking about those jobs, did you work at any of them for three months or more?’, and ‘Of the jobs you had for three months or more, did you leave (either/any) of them for a reason related to your accident?’.*

### Statistical analyses

Descriptive statistics including frequency distributions and measures of central tendency was undertaken to examine the distribution of key variables. Inferential statistics including chi-squares, t-tests and anovas testing were conducted to examine differences among key variables for each outcome. For SF12 V2, 173 participants (10.5 % of the study population) had missing values for at least 1 item. Those who had more than 4 items with missing values (*n* = 12) were excluded. Chi-square tests were conducted to test for differences between those with missing SF12 V2 values and those without missing SF12 V2 values. With the exception of gender, (*p* = .01) and age, (*p* < .001), there were no differences in the key personal characteristics or injury-related variables between those with missing values and those without missing values. Those without missing values were most likely to be male and younger than those with missing values. Therefore, missing data for the SF12 V2 questions were replaced by mean substitution [34].

Multiple linear regression models were used to determine the association between social support and physical health outcome (PCS). Multiple logistic regression models were used to estimate adjusted odds ratios (OR) with 95 % confidence intervals (CI) in relation to persistent pain and RTW outcomes. These models fit were evaluated with the Hosmer-Lemeshow goodness-of-fit tests. If necessary, non-significant variables were removed from the model when they interfered with the model fit. For each of the outcomes, 6 models were developed according to the variables of interest: family structure and sources of social support. For family structure, separate analyses were conducted for 1) marital status, 2) children and 3) family composition, while controlling for the effects of demographics and injury-related variables. For sources of social support, separate analyses were performed for 4) family, 5) friends and 6) neighbours’ support, while adjusting for the effects of family composition, demographics and injury-related variables. For the sources of social support variables, the *‘not often’* category was used as the reference group instead of the *‘no, not at all’* category as participants who rated not receiving any support may not be a homogenous group (e.g., participants who did not require any help, or did not have family living in the area). As a statistical association between physical health and social support was expected, regardless of the injury (i.e., being married is generally associated with better health outcomes [36]), these models were stratified by hospitalisation (hospitalised vs. non-hospitalised groups). If the association between health and social support were to be unrelated to the injury, one would expect the association to be similar in the hospitalised and non-hospitalised groups. Alternatively, if the association is stronger among hospitalised participants (i.e., more severely injured group), this would provide evidence to support an association between social support and injury outcomes. For persistent pain and RTW outcomes, the models were restricted to participants that were at least 3 months post-injury (*n* = 1026) and those who were employed at the time of accident (*n* = 955), respectively. Additional analysis was also conducted to explore the effects of employer support on RTW. In addition, in all models, interactions effects between social support and gender were explored as women and men may differ in the way they perceived social support [37, 38]. An interaction term with a p-value of less than .1 was considered sufficient to justify gender stratification.

Additional analyses of social support and time since injury were conducted to address the possibility of poor injury recovery leading to a deterioration of the social support network (reverse causality). For example, persons in poor health were more likely to have inadequate support and negative assessments of the support they received compared to healthier persons. Based on this assumption, it would be expected that social support would deteriorate during injury recovery; thus, an association between social support and time since injury would suggest that reverse causality was plausible. Alternatively, if results demonstrate that social support is not associated with time since injury, reverse causality would seem unlikely. Although the surveys were cross-sectional, the participants’ time since injury ranged from approximately 4 months up to 6 years. Therefore, the associations between time since injury (categorised as 0–12 months, 13–24 months, 25–36 months and 37+ months) and social support (categorised as *‘yes definitely’, ‘sometimes’, ‘not often’* and *‘no, not at all’*) were tested using chi-square tests. These were conducted on the total sample and separate analyses were also performed for the following subgroups: 1) participants who were hospitalised, 2) participants with persistent pain, and 3) participants that have not RTW. A p-value of less than .05 was considered significant in all analyses. Data analyses were conducted using the Statistical Analysis System (SAS) version 9.4. Ethical approval for this study was obtained from the host University Human Research Ethics Committee.

## Results

### Participant characteristics

The characteristics of the study population are presented in Table 1. The mean age of the cohort was 44 years (SD = 15). Over half of the participants were male (59 %), married or in a de facto relationship (54 %), and had children (56 %). Three quarters of the participants did not have a university level education (76 %) and were born in Australia (75 %). The majority were employed at the time of the accident (80 %). The most common occupations were technicians and trade workers (22 %), followed by professionals (18 %) and community/personal service workers (13 %). Over half were hospitalised (58 %) and had fractures (57 %) following a transport accident. Forty-three percent of the participants rated their health as excellent prior to the accident.

**Table 1:** Demographic characteristics of the sample

	N (column %)	Persistent pain^a^		RTW^b^		PCS
	(n = 1649)	N (row %)		N (row %)		Means (SD)
		Yes	No	Yes	No	(n = 1637)
		(n = 1026)	(n = 609)	(n = 955)	(n = 327)	
Gender						
Male	965 (58.5 %)	596 (62.3 %)	361 (37.7 %)	609 (74.7 %)	206 (25.3 %)	41.77 (7.03)
Female	684 (41.5 %)	430 (63.4 %)	248 (36.6 %)	346 (74.1 %)	121 (25.9 %)	40.79 (7.50)
p-value		0.64		0.80		.01
Age group						
16–24	176 (10.7 %)	89 (50.9 %)	86 (49.1 %)	116 (80.0 %)	29 (20.0 %)	43.67 (6.22)
25–34	307 (18.6 %)	183 (59.6 %)	124 (40.4 %)	225 (81.2 %)	52 (18.8 %)	42.87 (6.27)
35–44	365 (22.1 %)	237 (65.5 %)	125 (34.5 %)	238 (75.6 %)	77 (24.4 %)	42.47 (7.28)
45–54	392 (23.8 %)	275 (70.7 %)	114 (29.3 %)	222 (68.7 %)	101 (31.3 %)	40.11 (7.31)
55–64	247 (15.0 %)	148 (61.4 %)	93 (38.6 %)	130 (71.8 %)	51 (28.2 %)	40.82 (7.13)
65+	149 (9.0 %)	85 (57.4 %)	63 (42.6 %)	15 (48.4 %)	16 (51.6 %)	36.97 (7.62)
Missing	13 (0.8 %)	9 (69.2 %)	4 (30.8 %)	9 (90.0 %)	1 (10.0 %)	40.70 (8.49)
p-value		<.001		<.001		<.001
Marital status						
Married or in de facto relationship	896 (54.3 %)	568 (64.0 %)	319 (36.0 %)	561 (77.1 %)	167 (22.9 %)	41.62 (7.17)
Widowed/separated/divorced	284 (17.2 %)	197 (70.1 %)	84 (29.9 %)	101 (62.0 %)	62 (38.0 %)	39.63 (7.46)
Never married	459 (27.8 %)	254 (55.6 %)	203 (44.4 %)	289 (75.7 %)	93 (24.4 %)	41.91 (7.14)
Missing	10 (0.6 %)	7 (70.0 %)	3 (30.0 %)	4 (44.4 %)	5 (55.6 %)	42.01 (6.67)
p-value		<.001		<.001		<.001
Children						
Yes	918 (55.7 %)	574 (63.3 %)	333 (36.7 %)	558 (74.7 %)	189 (25.3 %)	41.71 (7.20)
No	717 (43.5 %)	444 (62.2 %)	270 (37.8 %)	391 (74.5 %)	134 (25.5 %)	40.89 (7.31)
Missing	14 (0.8 %)	8 (57.1 %)	6 (42.9 %)	6 (60.0 %)	4 (40.0 %)	42.65 (5.64)
p-value		0.65		0.93		.02
Family composition						
Married or in de facto relationship with children	511 (31.0 %)	332 (65.7 %)	173 (34.3 %)	332 (76.9 %)	100 (23.2 %)	41.87 (7.11)
Married or in de facto with no children	382 (23.2 %)	233 (61.5 %)	146 (38.5 %)	228 (77.6 %)	66 (22.5 %)	41.31 (7.25)
Widowed/separated/divorced with children	129 (7.8 %)	91 (72.2 %)	35 (27.8 %)	52 (64.2 %)	29 (35.8 %)	39.51 (7.41)
Widowed/separated/divorced with no children	154 (9.3 %)	105 (68.2 %)	49 (31.8 %)	49 (59.8 %)	33 (40.2 %)	39.72 (7.55)
Never married with children	276 (16.7 %)	149 (54.4 %)	125 (45.6 %)	173 (74.6 %)	59 (25.4 %)	42.43 (7.09)
Never married with no children	176 (10.7 %)	103 (58.5 %)	73 (41.5 %)	112 (77.2 %)	33 (22.8 %)	41.08 (7.22)
Missing	21 (1.3 %)	13 (61.9 %)	8 (38.1 %)	9 (56.3 %)	7 (43.8 %)	41.81 (5.89)
p-value		.002		.004		<.001
Educational level						
University level education	373 (22.6 %)	205 (55.9 %)	162 (44.1 %)	251 (83.7 %)	49 (16.3 %)	42.85 (7.00)
Less than University level education	1252 (75.9 %)	804 (64.6 %)	440 (35.4 %)	696 (71.6 %)	276 (28.4 %)	40.93 (7.29)
Missing	24 (1.5 %)	17 (70.8 %)	7 (29.2 %)	8 (80.0 %)	2 (20.0 %)	41.00 (5.50)
p-value		.002		<.001		<.001
Country of birth						
Australia	1243 (75.4 %)	769 (62.3 %)	466 (37.7 %)	752 (74.8 %)	253 (25.2 %)	41.55 (7.30)
Others	397 (24.1 %)	253 (64.7 %)	138 (35.3 %)	196 (72.6 %)	74 (27.4 %)	40.74 (7.05)
Missing	9 (0.5 %)	4 (44.4 %)	5 (55.6 %)	7 (70.0 %)	0 (0.0 %)	41.97 (6.48)
p-value		.38		.46		.05
Residential location						
Melbourne	1122 (68.0 %)	692 (62.3 %)	419 (37.7 %)	670 (76.8 %)	203 (23.3 %)	41.59 (7.08)
Rest of Victoria	459 (27.8 %)	285 (62.5 %)	171 (37.5 %)	249 (69.6 %)	109 (30.5 %)	41.10 (7.48)
All other	68 (4.1 %)	49 (72.1 %)	19 (28.0 %)	36 (70.6 %)	15 (29.4 %)	39.34 (8.04)
p-value		.27		.03		.22
Employed at the time of accident						
Yes	1320 (80.0 %)	815 (62.4 %)	491 (37.6 %)	955 (74.5 %)	327 (25.5 %)	41.93 (7.06)
No	325 (19.7 %)	208 (64.0 %)	117 (36.0 %)	0 (0.0 %)	0 (0.0 %)	39.07 (7.59)
Missing	4 (0.2 %)	3 (75.0 %)	1 (25.0 %)	0 (0.0 %)	0 (0.0 %)	38.34 (2.59)
p-value		.29				<.001
Occupation^c^						
Managers	136 (10.3 %)	83 (62.9 %)	49 (37.1 %)	98 (74.8 %)	33 (25.2 %)	41.62 (7.34)
Professionals	233 (17.7 %)	134 (58.8 %)	94 (41.2 %)	186 (84.9 %)	33 (15.1 %)	42.63 (6.95)
Technicians and trade workers	293 (22.2 %)	191 (65.4 %)	101 (34.6 %)	212 (73.4 %)	77 (26.6 %)	42.30 (6.99)
Community/personal service workers	166 (12.6 %)	95 (58.3 %)	68 (41.7 %)	113 (70.6 %)	47 (29.4 %)	41.92 (7.44)
Clerical/administration workers	132 (10.0 %)	91 (69.0 %)	41 (31.1 %)	103 (79.8 %)	26 (20.2 %)	40.51 (7.34)
Sales workers	95 (7.2 %)	60 (63.2 %)	35 (36.8 %)	73 (80.2 %)	18 (19.8 %)	42.74 (6.89)
Machine operators/drivers	100 (7.6 %)	54 (54.0 %)	46 (46.0 %)	70 (70.0 %)	30 (30.0 %)	41.15 (6.98)
Labourers	158 (12.0 %)	104 (66.2 %)	53 (33.8 %)	97 (61.8 %)	60 (38.2 %)	41.69 (6.56)
Missing	7 (0.5 %)	3 (42.9 %)	4 (57.1 %)	3 (50.0 %)	3 (50.0 %)	41.36 (5.67)
p-value		.19		<.001		.13
Income^c^						
Less than $50,000	526 (39.8 %)	333 (63.9 %)	188 (36.1 %)	370 (72.3 %)	142 (27.7 %)	41.74 (7.10)
More than $50,000	560 (42.4 %)	340 (61.6 %)	212 (38.4 %)	435 (80.1 %)	108 (19.9 %)	42.28 (6.87)
Missing	234 (17.7 %)	142 (60.9 %)	91 (39.1 %)	150 (66.1 %)	77 (33.9 %)	41.51 (7.41)
p-value		.43		.003		.20
Injury types						
Dislocation	119 (7.2 %)	68 (57.1 %)	51 (42.9 %)	79 (79.0 %)	21 (21.0 %)	42.80 (6.46)
Fracture	932 (56.5 %)	565 (61.0 %)	361 (39.0 %)	592 (77.7 %)	170 (13.3 %)	41.39 (7.36)
Soft tissue	517 (31.4 %)	344 (67.5 %)	166 (32.6 %)	246 (67.4 %)	119 (32.6 %)	41.09 (7.26)
Sprain/strain	81 (4.9 %)	49 (61.3 %)	31 (38.8 %)	38 (69.1 %)	17 (30.9 %)	40.63 (6.78)
p-value		.05		.001		.10
Hospitalisation (within 7 days of accident)						
Yes	953 (57.8 %)	597 (63.0 %)	351 (37.0 %)	561 (73.8 %)	199 (26.2 %)	41.14 (7.38)
No	696 (42.2 %)	429 (62.5 %)	258 (37.6 %)	394 (75.5 %)	128 (24.5 %)	41.67 (7.06)
p-value		.83		.50		.15
Health prior to accident						
Excellent	704 (42.7 %)	454 (65.0 %)	245 (35.1 %)	454 (76.3 %)	141 (23.7 %)	42.27 (7.12)
Very good	643 (39.0 %)	390 (61.2 %)	247 (38.8 %)	387 (75.0 %)	129 (25.0 %)	41.16 (7.03)
Good	241 (14.6 %)	148 (62.2 %)	90 (37.8 %)	99 (66.9 %)	49 (33.1 %)	40.34 (7.28)
Fair	46 (2.8 %)	26 (56.5 %)	20 (43.5 %)	13 (68.4 %)	6 (31.6 %)	36.59 (8.83)
Poor	13 (0.8 %)	6 (46.2 %)	7 (53.9 %)	1 (33.3 %)	2 (66.7 %)	38.35 (9.12)
Missing	2 (0.1 %)	2 (100.0 %)	0 (0.0 %)	1 (100.0 %)	0 (0.0 %)	40.06 (3.50)
p-value		.35		.07		<.001
Time post-injury						
0–12 months	368 (22.3 %)	222 (62.7 %)	132 (37.3 %)	208 (68.9 %)	94 (31.1 %)	41.51 (7.44)
13–24 months	561 (34.0 %)	341 (60.8 %)	220 (39.2 %)	335 (77.9 %)	95 (22.1 %)	41.91 (7.17)
25–36 months	377 (22.9 %)	211 (56.0 %)	166 (44.0 %)	221 (75.9 %)	70 (24.1 %)	41.75 (7.07)
37+ months	343 (20.8 %)	252 (73.5 %)	91 (26.5 %)	191 (73.7 %)	68 (26.3 %)	39.87 (7.19)
p-value		<.001		.05		<.001

^a^Restricted to those who were at least 3 months post-injury

^b,c^Restricted to those who were employed at the time of the accident

Table 1 presents the demographics of the study population and the demographics across the 3 outcomes: persistent pain, RTW and mean PCS score. Sixty-three percent of participants reported persistent pain and 74 % of participants have RTW. The mean PCS score was 41.36 (SD = 7.25). Males scored highly on the PCS compared to females (*p* = .01). Younger participants had a higher PCS score (*p* < .001) and were more likely to have RTW (*p* < .001). Older participants were more likely to experience persistent pain (*p* < .001). Participants who were widowed, separated, or divorced scored poorly on the PCS (*p* < .001), were less likely to have RTW (*p* < .001), and were more likely to experience persistent pain (*p* < .001) compared to those who were married or in a de facto relationship and never married. Participants with children had a higher PCS score compared to those with no children (*p* = .02). Participants with a university level education scored highly on the PCS (*p* < .001) and were more likely to have RTW (*p* < .001) than those without a university level education. They were also less likely to report persistent pain (*p* = .002). Participants at the 37+ months post–injury scored lower on the PCS (*p* < .001) and were also more likely to report persistent pain (*p* < .001) than participants at less than 36 months post-injury. Participants living in Melbourne (*p* = .03), employed as professionals (*p* < .001), earning more than $50,000 (*p* = .003), sustained a dislocation (*p* = .001) were more likely to have RTW.

### Social support and physical health

Table 2 presents the results of the multiple linear regression analyses examining the relationship between social support and physical health (PCS). Among the hospitalised participants, this table shows that no significant associations were found between marital status, children, family composition and PCS. On the other hand, receiving *‘definite’* family and friends’ support was significantly associated with an increase in PCS score.

**Table 2:** Multiple linear regression models of the relation between social support and physical component score stratified by hospitalisation

	Physical Component Score							
	Hospitalised				Non-hospitalised			
Models	Median	IQR	β	95 % CI	Median	IQR	β	95 % CI
1. Marital status^a^								
Married or in de facto relationship	44.15	37.91–48.07	0.98	−0.23–2.20	45.03	39.68–49.18	1.78*	0.38–3.19
Widowed/separated/divorced	41.79	36.85–47.29	0.87	−0.81–2.55	41.18	36.80–47.16	0.63	−1.14–2.40
Never married (ref)	44.50	38.98–49.27	0.00	.	46.21	38.94–50.50	0.00	.
2. Children^a^								
Yes	44.08	38.88–48.29	0.24	−0.77–1.24	44.95	39.09–49.28	−0.12	−1.20–0.96
No (ref)	43.78	37.55–48.11	0.00	.	44.48	37.98–48.71	0.00	.
3. Family composition^a^								
Married or in de facto relationship with children	44.13	38.65–48.07	1.04	−0.50–2.58	45.21	39.61–49.28	2.14*	0.16–4.11
Married or in de facto with no children	44.20	37.43–48.21	0.93	−0.67–2.53	45.02	39.68–48.71	2.88*	0.85–4.90
Widowed/separated/divorced with children	41.21	36.37–45.56	0.24	−1.95–2.42	42.90	37.60–47.88	1.35	−1.08–3.78
Widowed/separated/divorced with no children	42.56	37.40–49.45	1.05	−0.97–3.07	40.09	36.47–46.88	1.26	−1.24–3.78
Never married with children	45.17	40.22–49.82	0.13	−1.57–1.83	46.03	38.71–50.50	1.16	−0.91–3.22
Never married with no children (ref)	43.78	37.73–47.96	0.00	.	46.27	38.94–50.53	0.00	.
4. Family^b^								
Definitely	45.09	39.56–49.19	3.68*	1.56–5.80	45.83	40.11–49.44	0.58	−1.46–2.62
Yes, sometimes	41.19	36.24–46.73	1.72	−0.57–4.01	42.94	37.37–48.99	−0.73	−2.99–1.53
No, not at all	42.54	36.44–47.64	2.48	−0.15–5.10	40.04	35.96–46.14	−2.56*	−5.04–−0.08
Not often (ref)	40.68	37.46–46.63	0.00	.	42.67	38.58–47.51	0.00	.
5. Friends^b^								
Definitely	45.25	39.26–49.02	2.68*	0.80–4.56	46.21	40.61–49.77	0.56	−1.51–2.64
Yes, sometimes	42.66	37.43–47.77	0.46	−1.52–2.43	44.01	39.18–49.47	−0.70	−2.88–1.49
No, not at all	40.39	35.30–45.61	0.07	−2.40–2.54	38.86	35.76–43.34	−3.35*	−5.92– −0.78
Not often (ref)	41.73	37.59–47.31	0.00	.	42.37	37.96–47.51	0.00	.
6. Neighbours^b^								
Definitely	45.31	40.33–49.03	1.44	−0.17–3.05	46.53	41.68–49.78	1.39	−0.33–3.11
Yes, sometimes	43.54	38.35–48.07	0.24	−1.42–1.91	45.85	38.71–50.56	−0.29	−2.13–1.55
No, not at all	42.86	37.07–47.96	−0.57	−2.12–0.97	42.70	37.44–47.96	−1.86*	−3.48– −0.23
Not often (ref)	43.66	37.59–49.18	0.00	.	44.65	38.47–49.28	0.00	.

^a^All models adjusted for gender, age, education, country of birth, residential location, injury types, prior health and days post-injury

^b^All models adjusted for family composition, gender, age, education, country of birth, residential location, injury types, prior health and days post-injury

*p < .05

Among non-hospitalised participants, being married or in a de facto relationship was significantly associated with an increase in PCS score. In addition, non-hospitalised participants who reported having ‘*no, not at all’* support from family, friends, and neighbours were significantly associated with a poor score on the PCS (Table 2). In addition, no interactions effects were observed for gender and physical health.

### Social support and persistent pain

Table 3 presents the results of the multiple logistic regression analyses investigating the relationship between social support and persistent pain. The table shows that being widowed, separated or divorced was significantly associated with increased odds of reporting persistent pain. Participants who reported having *‘definite’* support from family, friends and neighbours, relative to those reporting ‘*not often’* support were significantly associated with decreased odds of reporting persistent pain. No statistically significant associations with persistent pain were observed in the family structure models for children and family composition. To test if these results were due to a biased sample selection (i.e., participants who have RTW may be more positive about their experience of persistent pain), a separate model was conducted for participants who were working at the time of the accident (not shown). Results from the model with only participants working at the time of the accident were similar to the previous models, thus, results were unlikely due to the sample selection bias. In addition, no interaction effects were observed for gender and persistent pain.

**Table 3:** Multiple logistic regression models of the relation between social support and persistent pain

	Persistent pain^a^	
	OR	95 % CI
Models		
1. Marital status^b^		
Married or in de facto relationship	1.28	0.97–1.69
Widowed/separated/divorced	1.62*	1.11–2.37
Never married (ref)		
Hosmer-Lemeshow fit test	*X* ^2^(8) = 11.36 *p* = 0.18	
2. Children^b^		
Yes	0.96	0.77–1.20
No (ref)		
Hosmer-Lemeshow fit test	*X* ^2^(8) = 3.12 *p* = 0.92	
3. Family composition^b^		
Married or in de facto relationship with children	1.14	0.78–1.68
Married or in de facto with no children	1.13	0.76–1.68
Widowed/separated/divorced with children	1.49	0.87–2.55
Widowed/separated/divorced with no children	1.41	0.85–2.33
Never married with children	0.84	0.55–1.26
Never married with no children (ref)		
Hosmer-Lemeshow fit test	*X* ^2^(8) = 10.27 *p* = 0.25	
4. Family^c^		
Definitely	0.40*	0.24–0.68
Yes, sometimes	0.68	0.39–1.19
No, not at all	0.58	0.31–1.07
Not often (ref)		
Hosmer-Lemeshow fit test	*X* ^2^(8) = 9.95 *p* = 0.26	
5. Friends^d^		
Definitely	0.29*	0.17–0.47
Yes, sometimes	0.50*	0.30–0.89
No, not at all	1.14	0.58–2.25
Not often (ref)		
Hosmer-Lemeshow fit test	*X* ^2^(8) = 12.23 *p* = 0.14	
6. Neighbours^c^		
Definitely	0.59*	0.41–0.84
Yes, sometimes	0.78	0.54–1.15
No, not at all	1.27	0.89–1.81
Not often (ref)		
Hosmer-Lemeshow fit test	*X* ^2^(8) = 5.39 *p* = 0.72	

^a^Restricted to those who were at least 3 months post-injury

^b^All models adjusted for gender, age, education, country of birth, residential location, injury types, prior health, days post-injury and hospitalisation

^c^All models adjusted for family composition, gender, age, education, country of birth, residential location, injury types, prior health, days post-injury and hospitalisation

^d^Due to problem with model fit, non-significant variables were removed. The final model was adjusted for age, education and days post-injury

*p < .05

### Social support and RTW

Interaction effects were observed for gender and RTW (p < .1); thus the RTW models were further stratified by gender. Table 4 presents the results of the multiple logistic regression models assessing the relationship between social support and RTW. The table shows that significant differences were found between men and women. Women receiving support from family, regardless of the amount of support, had decreased odds for RTW, whereas *‘definite’* support from friends increased the odds of RTW. No such differences were observed among men. For both men and women, support from employers was positively associated with RTW. The associations were more prominent among women. No significant associations were found for marital status and children for both groups.

**Table 4:** Multiple logistic regression models of the relation between social support and RTW, stratified by gender

	RTW ^a^			
	Men		Women	
Models	OR	95 % CI	OR	95 % CI
1. Marital status^b^				
Married or in de facto relationship	1.56	0.92–2.64	0.72	0.33–1.57
Widowed/separated/divorced	0.66	0.31–1.37	0.67	0.26–1.71
Never married				
Hosmer-Lemeshow fit test	*X* ^2^(8) = 6.98 *p* = 0.54		*X* ^2^(8) = 2.13 *p* = 0.98	
2. Children^b^				
Yes	0.84	0.55–1.27	0.94	0.53–1.67
No (ref)				
Hosmer-Lemeshow fit test	*X* ^2^(8) = 14.53 *p* = 0.07		*X* ^2^(8) = 8.03 *p* = 0.43	
3. Family composition^b^				
Married or in de facto relationship with children	1.22	0.58–2.58	0.57	0.20–1.63
Married or in de facto with no children	1.24	0.56–2.77	0.77	0.26–2.29
Widowed/separated/divorced with children	0.28*	0.10–0.79	0.84	0.24–2.89
Widowed/separated/divorced with no children	0.88	0.31–2.50	0.38	0.10–1.39
Never married with children	0.64	0.28–1.48	0.82	0.27–2.49
Never married with no children (ref)				
Hosmer-Lemeshow fit test	*X* ^2^(8) = 3.24 *p* = 0.92		*X* ^2^(8) = 8.22 *p* = 0.41	
4. Family^c^				
Definitely	2.06	0.76–5.57	0.09*	0.01–0.78
Yes, sometimes	1.35	0.46–3.95	0.07*	0.01–0.62
No, not at all	0.91	0.28–2.93	0.07*	0.01–0.72
Not often (ref)				
Hosmer-Lemeshow fit test	*X* ^2^(8) = 8 *p* = 0.75		*X* ^2^(8) = 12.95 *p* = 0.11	
5. Friends^c^				
Definitely	1.09	0.38–3.10	3.03*	1.15–8.02
Yes, sometimes	0.45	0.16–1.31	2.18	0.77–6.12
No, not at all	0.42	0.12–1.51	1.37	0.36–5.22
Not often (ref)				
Hosmer-Lemeshow fit test	*X* ^2^(8) = 9.85 *p* = 0.28		*X* ^2^(8) = 9.81 *p* = 0.28	
6. Neighbours^c^				
Definitely	1.89	0.90–3.97	1.24	0.43–3.60
Yes, sometimes	1.05	0.51–2.16	0.69	0.24–2.01
No, not at all	0.49*	0.25–0.96	0.65	0.24–1.74
Not often (ref)				
Hosmer-Lemeshow fit test	*X* ^2^(8) = 5.25 *p* = 0.73		*X* ^2^(8) = 11.71 *p* = 0.16	
7. Employers^c^				
Very supportive	5.62*	2.77–11.38	7.22*	2.58–20.20
Somewhat supportive	4.35*	1.90–9.95	6.87*	1.86–25.23
A little supportive	2.86*	1.05–7.76	1.72	0.44–6.79
Not at all supportive (ref)				
Hosmer-Lemeshow fit test	*X* ^2^(8) = 7.50 *p* = 0.48		*X* ^2^(8) = 5.15 *p* = 0.74	

^a^Restricted to those who were employed at the time of the accident

^b^All models adjusted for age, education, country of birth, residential location, injury types, prior health, days post-injury, hospitalisation, income and occupation

^c^All models adjusted for family composition, age, education, country of birth, residential location, injury types, prior health, days post-injury, hospitalisation, income and occupation

*p < .05

### Reverse causality

**Fig. 1:**
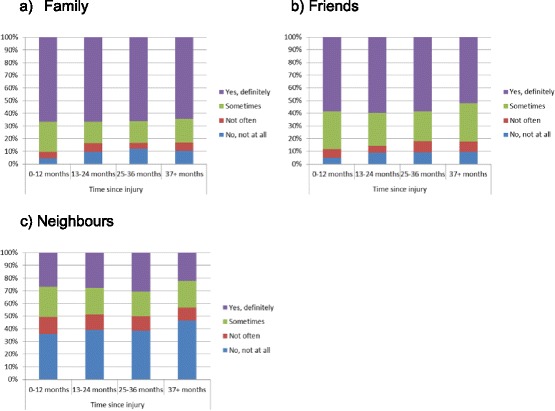
The relation between the source of social support and time since injury for the total sample

**Fig. 2:**
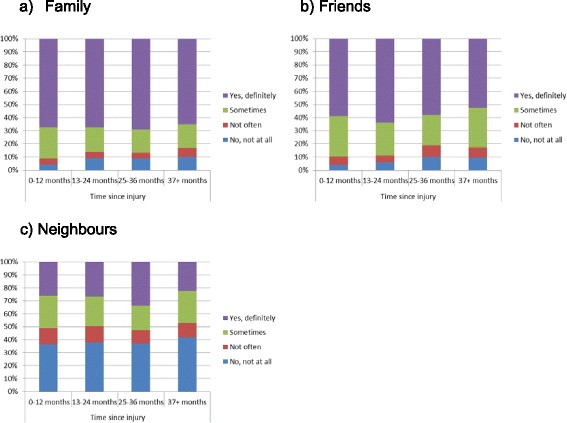
The relation between the source of social support and time since injury among hospitalised participants

**Fig. 3:**
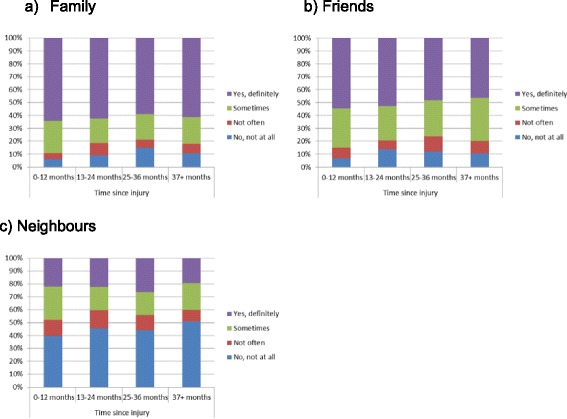
The relation between the source of social support and time since injury among participants with persistent pain

**Fig. 4:**
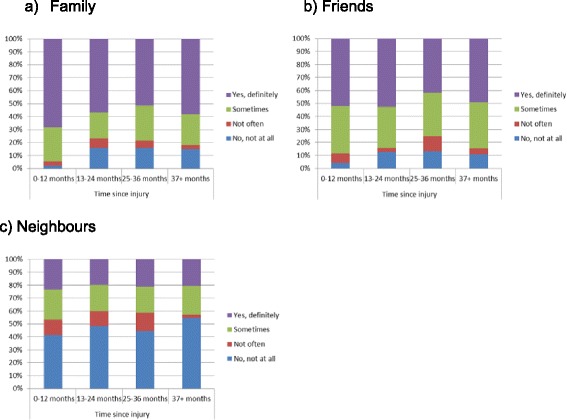
The relation between the source of social support and time since injury among participants who did not RTW

To ascertain the possibility of reverse causality, chi-square tests were conducted between time since injury and the various sources of social support (family, friends and neighbours). The models showed a significant association between time since injury and perceived level of family support*.* Participants at the 0–12 months post-injury were more likely to report having ‘*yes, definitely’*, and ‘*sometimes*’ support from family and less likely to report having *‘no, not all’* support from family compared to participants at the 13 months or more post-injury (*X*^2^(9, *N* = 1642) = 21.90, *p* < .01) (Fig. 1a). A similar pattern was observed among hospitalised participants for family’s support, but the statistical association was not significant (*X*^2^(9, *N* = 948) = 10.27, *p* = .32) (Fig. 2a). No statistical associations were found between friends, neighbours’ support and time since injury for the total sample (Fig. 1b & c) and the hospitalised participants (Fig. 2b & c). In addition, no statistical associations were found between family, friends and neighbours’ support and time since injury for participants with persistent pain (Fig. 3a, b & c) and participants who did not RTW (Fig. 4a, b & c).

## Discussion

The present study examined the relationship between social support and injury outcomes among people with MSI sustained in a transport accident. The results revealed that several aspects of the family structure and sources of social support had a positive impact on physical health, persistent pain and RTW. This study also showed significant gender differences in RTW. The strength of the associations between social support and each of the outcomes varied across family structure and sources of support.

The results of this study can be interpreted using the convoy model. The convoy model suggests that intimate and confiding relationships are regarded as the most beneficial effects of social support [39, 40] and that the availability of a spouse to provide support appears to buffer against the impact of stress from injury [28]. The findings of this study found support for this relationship. The data indicated that recovery is influenced by the family structure, sources of social support and the severity of the injury. Among hospitalised persons, strong support from family and friends was associated with better physical health. Non-hospitalised persons who reported having no or little support from family and friends had poor physical health scores. In support, past research has shown that severe injury from transport accident requires substantial support from family members [13] and that a lack of social support in general resulted in higher rates of complications [27]. The perception of no or low support could also potentially reflect difficulties in reaching out to support networks or difficulties between asserting independence and being supported by others [13]. Among non-hospitalised persons, family structure such as being married or in a de facto relationship, with or without children, was associated with better physical health. Marriage may be a factor reducing the need for hospitalisation, with several studies demonstrating that married individuals have decreased risk of being hospitalised for a number of diseases [41, 42].

Consistent with the convoy model [28], the results demonstrated that the source of social support followed a hierarchically descending order from family to friends among hospitalised persons. Closer and more stable circles (i.e., family) compared with less close and stable circles (i.e., friends and neighbours) were viewed more favourably for support. As family relationships are bonded by intimacy and kinship, they tend to be more stable compared with friends and therefore are more likely to be the preferred source of support [43]. According to the convoy model, different network members also serve different functions [28]. Past research has shown that family members are often core providers of material aid and instrumental support, whereas friends more often provide emotional support and companionship [43]. Instrumental support may be the preferred type of support for the improvement of physical health, and this support may have been more likely to be provided by family members than friends. However, we were unable to assess whether the significant association between family support and physical health was related to a specific type of support. Further research is warranted to distinguish among the types of support provided by the different sources of support.

According to the convoy model, people who are separated or divorced tend to lose their social network members [28]. For example, people may have less contact with their in-laws and friends to avoid a situation of ‘taking sides’. In contrast, widowhood may lead to an increase in social support in the initial period after the loss of a spouse but the level of support may decrease over time [44]. We found that persons, who were widowed, separated or divorced were almost 2 times as likely to report persistent pain compared to those who never married. This finding has also been supported in the research literature. Persons who are widowed, separated or divorced tend to have worst physical and mental health than those who are married or never married [45]. Widowhood, separation or divorce has also been found to increase the risk of social isolation [46]. These findings suggest that the lack of social support to buffer the effects of pain may contribute to the presence of self-reported persistent pain. In further support of this argument, we found that receiving strong support from family, friends and neighbours were negatively associated with persistent pain. This is consistent with previous pain studies [47–49] which suggest that people who have access to a large support network, who seek social comfort, understanding and share their concern with others, manage their pain in a more adaptive manner.

Inconsistent with the convoy model, the results showed that, compared to those who reported not receiving frequent support, the odds of reporting persistent pain were reduced for those with strong support from friends (71 % lower), family (60 % lower), and neighbours (41 % lower). The results suggested that friends’ support was the preferred source over family and neighbours’ support in reporting the absence of persistent pain. This result suggests that friends and family members offer different types of support in situations of persistent pain. In support, research has shown that family members have been found to be responsible for providing day-to-day physical assistance whereas friends are more likely to offer social comfort for pain management [50]. However, as previously mentioned, we were unable to assess whether the significant association between each source of support and persistent pain was related to a specific type of support.

According to the convoy model, social support and social relations are shaped by personal characteristics including gender [28]. Gender can alter the adequacy of support provided by the convoy and subsequently leads to a change in its structure and function. Our results showed some support for this finding, in regards to variation in gender for RTW outcome. Interestingly, we found women receiving support from family, regardless of the amount received, had decreased odds for RTW. Potential reasons for this finding may be that women were less inclined to RTW if they had an alternative source of income such as financial support from their spouse or family members. Alternatively, support from family may negatively influence the recovery process. Strong support such as pushing for recovery, being too protective or helpful may actually have the opposite effect. Women may feel distress, resentment or dependency towards their family members [27, 51].

Whilst support from family was negatively associated with RTW, we found that strong support from friends was positively associated with RTW among women. No such associations were observed among men. The gender differences found may be explained by socialisation. Men’s socialisation focuses on autonomy, self-reliance, independence, and de-emphasising feelings whereas for women, socialisation emphasises verbal expressiveness, warmth and intimacy [52]. In support, women have generally more close friends and develop more intimate interpersonal relationships than men [53]. Women are also more likely to seek help and to mobilise their support network than men in times of needs [39, 54, 55]. Although men usually have more extensive networks than women, men are likely to cite their spouse as their only confident, whereas women cite spouses and friends about the same frequency [56, 57].

We also found that for both men and women, support from employers was positively associated with RTW, although the association was stronger among women. This is consistent with previous studies which found that men benefit from employment support more than family or friends’ support [58] and that women received more support from supervisors than men [59]*.* Men have a strong attachment to work roles [60] and are more likely to RTW faster than women [61]. The stronger association between employment support among women may reflect differences in gender socialisation in which women are more likely than men to give and receive support [55]. An alternative explanation is that organisational policies such as affirmative action which encourage women’s RTW may explain the gender differences in RTW. A strong supportive work environment can benefit women by reducing stress, in turn increasing organisational commitment and decreasing absenteeism and turnover [62].

Although the study provides greater insight into the role of social support across a range of MSI outcomes, the results should be interpreted with regard to several study limitations. First, the cross-sectional design of the study limited interpretation of the exact nature of the relationship between social support and MSI outcomes. We attempted to control for hospitalisation (via stratification) in an effort to reduce the likelihood that the effects of social support are reflective of an association between health and social support that is unrelated to the MSI. The chi-square results also suggested low potential for reverse causality. Future longitudinal studies are required in order to establish causality and causal pathways. Second, a ‘good-old-days’ bias in self-report of pre-injury health status may be present with the majority of individuals rating their health as excellent or very good prior to the MSI which could have potentially resulted in an over-estimation of their pre-injury health and therefore leading to residual confounding. Future studies should assess pre-injury health status as soon after the injury as possible with a validated measure or obtained pre-injury medical records as a proxy of pre-injury health. Third, although we had information on the source of social support, the survey included a non-standardised measure of social support (single dimension) with limited information on the construct of support (different types of support). Future studies are required to assess the multi-dimensional constructs of support which will lead to a more complete understanding of the impact of social support on outcomes among individuals with MSI. Fourth, persistent pain was measured using a non-standardised measure of pain which may not accurately reflect the presence of persistent pain. Future studies should use a validated measure of persistent pain. Fifth, we did not have information on personality traits which could have potentially influence the perception of social support. For example, individuals with high self-esteem and great social skills are more likely to have a supportive network system than someone who has low self-esteem and poor social skills [63]. In addition, individuals who are confident may have preferred to be independent rather than rely on others for support. Future studies could incorporate a measurement of personality traits in the research design. Sixth, the study is limited by assessing only 1 perspective. As social support is a transaction between 2 or more people, the information might be biased by individual characteristics that filter perceptions. Further research is warranted to assess the interactions and effects of all persons involved in these supportive transactions. Finally, we acknowledge that the testing of a number of models may have increased the chance of a type 1 error. However, all of our hypotheses were developed a-priori and theoretically underpinned by the relevant research literature. Thus, adjusting the alpha level was not considered necessary [64–66].

In summary, the present study confirms the importance of family structure and source of social support in recovery among people with MSI sustained in a transport accident. This study also provides a practical application of the convoy model to an area that requires attention, given the extent of the problem. The study findings have implications for educating social networks members about their potential role in promoting recovery and informing interventions. When developing interventions aiming to strengthen the person’s support network, the structure of the network, the source of social support, type of outcomes, injury severity and gender need to be taken into consideration. In addition, this study also highlights the importance of identifying people who have limited access to a social support network. People with limited access to social support from sources such as family, friends and neighbours could potentially benefit from alternative sources of practical and psychological support, such as home services and counselling, respectively. The findings in this study encourage further research exploring alternative pathways to attaining support if unavailable from the social network, and determining subsequent impact on injury outcomes.
